# Effects of Lysophospholipid Supplementation in Feed with Low Protein or Lipid on Growth Performance, Lipid Metabolism, and Intestinal Flora of Largemouth Bass (*Micropterus salmoides*)

**DOI:** 10.1155/2022/4347466

**Published:** 2022-09-06

**Authors:** Ziye Lu, Chunfeng Yao, Beiping Tan, Xiaohui Dong, Qihui Yang, Hongyu Liu, Shuang Zhang, Shuyan Chi

**Affiliations:** ^1^Laboratory of Aquatic Animal Nutrition and Feed, College of Fisheries, Guangdong Ocean University, Zhanjiang, Guangdong, China; ^2^Guangdong Yuehai Feed Group Co., Ltd., Zhanjiang, Guangdong, China

## Abstract

The largemouth bass (*Micropterus salmoides*) were fed diets with three experimental feeds, a control diet (Control, crude protein (CP): 54.52%, crude lipid (CL): 11.45%), a low-protein diet with lysophospholipid (LP-Ly, CP: 52.46%, CL: 11.36%), and a low-lipid diet with lysophospholipid (LL-Ly, CP: 54.43%, CL: 10.19%), respectively. The LP-Ly and LL-Ly groups represented the addition of 1 g/kg of lysophospholipids in the low-protein and low-lipid groups, respectively. After a 64-day feeding trial, the experimental results showed that the growth performance, hepatosomatic index, and viscerosomatic index of largemouth bass in both the LP-Ly and LL-Ly groups were not significantly different compared to those in the Control group (*P* > 0.05). The condition factor and CP content of whole fish were significantly higher in the LP-Ly group than those in the Control group (*P* < 0.05). Compared with the Control group, the serum total cholesterol level and alanine aminotransferase enzyme activity were significantly lower in both the LP-Ly group and the LL-Ly group (*P* < 0.05). The protease and lipase activities in the liver and intestine of both group LL-Ly and group LP-Ly were significantly higher than those of the Control group (*P* < 0.05). Compared to both the LL-Ly group and the LP-Ly group, significantly lower liver enzyme activities and gene expression of fatty acid synthase, hormone-sensitive lipase, and carnitine palmitoyltransferase 1 were found in the Control group (*P* < 0.05). The addition of lysophospholipids increased the abundance of beneficial bacteria (*Cetobacterium* and *Acinetobacter*) and decreased the abundance of harmful bacteria (*Mycoplasma*) in the intestinal flora. In conclusion, the supplementation of lysophospholipids in low-protein or low-lipid diets had no negative effect on the growth performance of largemouth bass, but increased the activity of intestinal digestive enzymes, enhanced the hepatic lipid metabolism, promoted the protein deposition, and regulated the structure and diversity of the intestinal flora.

## 1. Introduction

The global aquafeed production in 2020 was 51.37 million tonnes, increasing by 3.7% compared with 2019 [1]. Higher dietary lipid and protein contents in aquafeeds not only increase farming cost but also lead to waste and ammonia emissions. However, lower dietary lipid or protein contents could result in negative effects on fish growth performance [2, 3]. Therefore, how to save cost and at the same time to maintain development quality has become an important and urgent issue. Improving the efficiency of nonprotein energy utilization is one of the ways to spare feed protein in aquafeeds.

Lipids have been effectively used to spare protein [4]. Feed additives like phospholipids (PL) have been used to enhance lipid utilization. The PL supplementation in diets of large yellow croaker (*Larmichthys crocea*) can improve the protein efficiency and protein deposition [5]. After ingestion of phospholipids, large yellow croaker showed increased body protein deposition, enhanced activities of trypsin and amylase, and improved development of digestive tract [6]. In hybrid grouper (*Epinephelus fuscoguttatus* ♀ × *E. lanceolatus* ♂), a significant increase in liver and whole-body crude protein content was observed when phospholipids were supplemented in the diet [7]. The addition of phospholipids to low fishmeal diets for mud crab (*Scylla paramamosain*) can significantly increase the protein efficiency and body crude protein content [8].

Lysophospholipid, degraded phospholipid by pancreatic phospholipase A_2_ [9, 10], not only increased the release of mono- and diglyceride fatty acid esters by emulsifying the lipid [11, 12] but also altered the membrane permeability, which could increase the pore area of intestinal cell membrane [13, 14] and improve the digestion and absorption of dietary fatty acids [15]. It is worth noting that lysophospholipid also plays a key role in a variety of cellular signaling mechanisms. In rats, it promoted the fatty acid catabolism through activation of the AMPK*α*-ACC-CPT signaling and mitogen-activated protein kinase (MAPK) signaling pathways [16–18]. In turbot, it reduced the hepatic lipid content, plasma triglyceride concentration, and total cholesterol level and increased the plasma free fatty acid contents as well [19]. However, the lysophospholipid synthesis in fish is usually insufficient to meet their metabolic requirements [20, 21]. Diets supplemented with exogenous lysophospholipid could improve protein efficiency ratio and growth of rainbow trout (*Oncorhynchus mykiss*) [22].

The largemouth bass (*Micropterus salmoides*) is a typical carnivorous fish species that relies on high level of dietary protein and lipid. Although carbohydrates are the cheapest energy source, this species cannot use carbohydrates efficiently, and high dietary carbohydrate levels often lead to abnormal sugar metabolism and liver dysfunction [23]. The objective of this experiment was to investigate whether three diets could satisfy the growth of largemouth bass and improve the utilization of dietary protein and lipid after reducing the crude protein or crude lipid content of the diets and supplementing with lysophospholipids.

## 2. Materials and Methods

### 2.1. Experimental Diets

Three diets were formulated with crude protein/crude lipid levels of 54.52%/11.45% (Control group), 52.46%/11.36% (LP group), and 54.43%/10.19% (LL group), respectively (Table 1). Lysophospholipid was added into the LP and LL diet at 1 g/kg, to obtain the lysophospholipid-supplemented groups, which was designated as LP-Ly and LL-Ly, respectively. The ingredients were smashed and passed through a 60-mesh sieve and then mixed for 15 minutes with a V-type vertical mixer (JS-14S type, Zhejiang China Electric Co., Ltd.). Oil and water were added into the mixture to form a paste that were put into the extruded machine to make the pellets with a diameter of 3.0 mm. The pellet feeds were dried at room temperature of 25°C with ventilation for 48 h and then sealed and stored at -20°C until use.

### 2.2. Experimental Fish and Feeding Trials

Largemouth bass juveniles were purchased from Zhenghe Fish and Shrimp Hatchery Co. Ltd. (Zhuhai, China), stocked in continuously aerated fiberglass tanks (1000 L), and fed commercial diets (0#, Rongchuan Co. Ltd., Zhuhai, China). After the fish had acclimatized to the experiment environment for 2 weeks, 270 healthy and uniformly sized juvenile largemouth bass (initial weight 6.04 ± 0.04 g) were randomly selected and divided into nine fiberglass buckets (300 L), after being fasted for 24 h. Each experimental group had three replicate tanks. The fish were fed the test feeds at 08:00 and 16:00 daily. The initial feeding rate was 3% according to body weight and then was adjusted to satiation feeding. The daily feed consumption and fish mortality in each experimental group were recorded. The feeding trial was carried out in an indoor hydrostatic system with aeration. The water conditions are as follows: temperature 29-32°C and dissolved oxygen >5 mg/L. The water was changed at 60-80% every day. The feeding trial lasted 9 weeks.

### 2.3. Sample Collection

At the end of 9 weeks of feeding trial, fish were fasted for 24 h. After anesthetized with eugenol (1 : 10000, Sinopharm Chemical Reagent Co., Ltd), fish in each tank were counted and weighed in order to calculate the weight gain rate (WGR), specific growth rate (SGR), feed conversion ratio (FCR), and survival rate (SR). Three largemouth bass were randomly collected. Their body weight and length as well as the liver and visceral mass weight were measured to calculate the condition factor (CF), hepatosomatic index (HSI), and viscerosomatic index (VSI).

### 2.4. Calculations and Statistical Analysis

Weight gain rate (WGR, %) = 100 × (final body weight, g − initial body weight, g)/(initial body weight, g).

Specific growth rate (SGR, %/d) = 100 × (Ln final body weight, g − Ln initial body weight, g)/feeding days.

Survival rate (SR, %) = 100 × final fish number/initial fish number.

Feed conversion ratio (FCR) = (dry weight of feed, g)/(final body weight, g − initial body weight, g).

Hepatosomatic index (HSI, %) = 100 × liver weight, g/body weight, g.

Viscerosomatic index (VSI, %) = 100 × viscera weight, g/body weight, g.

Condition factor (CF) = 100 × body weight, g/body length (cm)^3^.

Feeding ratio (FR, %) = 100 × dry feed consumed/[days × (final fish number + initial fish number)/2].

Protein deposition ratio (PDR, %) = 100 × [(final body weight (g) × crude protein of end fish (%) − initial body weight (g) × crude protein of the starting fish body)]/(feed protein intake (g) × crude protein of feed (%)).

All data were subjected to independent samples *t*-test using SPSS 21.0 statistical software, and descriptive statistics were expressed as mean ± standard error (SEM), with *P* < 0.05 indicating a significant difference.

### 2.5. Chemical Composition Analysis

The proximate composition of fish body and diets were measured according to the standard AOAC methods [24]. Moisture was assayed by drying the samples at 105°C to a constant weight. Crude protein (*N* × 6.25) and crude lipid were assayed with the Kjeldahl method (2300-Auto-analyzer, Foss, Sweden) and Soxhlet extraction, respectively. Ash was measured by incineration at 550°C in a muffle furnace.

### 2.6. Biochemical Parameters and Enzyme Activity

Tail vein blood was drawn from 6 fish in each tank, placed in 1.5 mL centrifuge tubes, left for 12 h, and then centrifuged (4000 r/min) for 10 min at 4°C. The serum was separated and stored at -80°C. The liver and intestine were dissected, rinsed with saline, and homogenized with PBS (pH 7.4) at ice bath. The samples were centrifuged for 20 min at 2500 r/min. The supernatants were stored at -80°C for enzyme activity assays.

Serum biochemical indicators were measured using commercial kits (Nanjing Jiancheng Institute of Biological Engineering Co. Ltd., China). Total protein (TP) (A045-2-2), triglycerides (TG) (A110-1-1), total cholesterol (TC) (A111-1-1), high-density lipoprotein cholesterol (HDL-C) (A112-1-1), low-density lipoprotein cholesterol (LDL-C) (A113-1-1), alanine aminotransferase (ALT) (C009-2-1), and aspartate aminotransferase (AST) (C010-2-1) in serum were measured using full-wavelength microplate reader (Thermo Scientific Multiskan GO, America) at 595 nm, 510 nm, 510 nm, 546 nm, 546 nm, 510 nm, 510 nm, and 510 nm, respectively.

Tissue enzyme activities were analyzed using the enzyme-linked immunosorbent assay (ELISA) kit (Shanghai Enzyme Linkage Biotechnology Co. China). The OD values were read for amylase (ml036449), lipase (ml036371), trypsin (ml064285), lipoprotein lipase (LPL) (ml036373), hormone-sensitive lipase (HSL) (ml036437), fatty acid synthase (FAS) (ml036370), acetyl coenzyme A carboxylase (ACC) (ml036379), and carnitine palmitoyltransferase 1 (CPT1) (ml036379) at 450 nm using a microplate reader (Rayto RT-6100, China), strictly following the kits' instructions.

### 2.7. Quantitative Real-Time PCR Analysis

Three fish were randomly selected from each tank and dissected. The liver was immersed in RNAlater (Ambion, USA) and then stored at -80°C for the mRNA expression assays. Total RNA of the samples were extracted using the commercial kit (Beijing All-Style Gold Biotechnology Co., Ltd.). The RNA quality was assessed with agarose gel electrophoresis and ultramicroscopic spectrophotometer (NanoDrop-1000, Wilmington, USA). The cDNA was obtained using the Prime Script™ RT reagent kit (Takara, Japan). Real-time fluorescent quantitative PCR (LightCycler480) was performed using SYBR® Green Master Mix (Takara, Japan). The 10 *μ*L reaction system consisted of 5 *μ*L 2× SYBR Green (Takara, Japan), 1 *μ*L cDNA, 0.5 *μ*L primers, and 3 *μ*L sterile double-distilled water. The processes were as follows: 95°C denaturation step for 30 s, 40 amplification cycles “denaturation at 95°C for 5 s, annealing at 60°C for 30 s,” followed by melt curve analysis and cooling to 4°C. The relative mRNA expression levels of the target genes were calculated according to equation 2^-*ΔΔ*Ct^ using *β-actin* as the housekeeping gene [25]. The primer sequences are presented in Table 2.

### 2.8. High-Throughput Sequencing and Processing

Two fish were randomly selected from each tank. The fish were wiped with 75% alcohol, and the intestines were taken and stored at -80°C until the intestinal flora was assayed. The gut microflora structure was analyzed with 16S rDNA sequencing by Guangzhou Genedenovo Biotechnology Co. Genomic DNA was extracted using the Magen Hipure Soil DNA Kit (Qiagen, Germany). The upstream primer is “CCTACGGGNGGCWGCAG” and the downstream primer is “GGACTACHVGGGTATCTAAT.” After the amplified product was purified (Monarch DNA Gel Extraction Kit, New England Biolabs Ltd., Beijing) and quantified, samples were mixed to a 1 : 1 mass ratio. Library construction and sequencing were performed on an Illumina HiSeq sequencing platform (HiSeq 2500, Illumina, USA). Finally, the alpha diversity index of the samples was calculated and analyzed using Mothur (version 1.3.0) software.

## 3. Results

### 3.1. Growth Performance

No significant differences were observed in final body weight (FBW), SGR, WGR, and SR between dietary groups (*P* > 0.05) (Table 3). The FCR of largemouth bass in groups LP-Ly and LL-Ly was 2% and 4% lower than that in the Control group, respectively (*P* > 0.05). No significant differences in HSI and VSI were found (*P* > 0.05). Nevertheless, the HSI was 9.52% and 6.12% lower in LP-Ly and LL-Ly compared to Control. The CF was significantly higher in the LP-Ly group than that in the Control group (*P* < 0.05).

### 3.2. Whole Fish Composition

There were no significant differences in moisture, crude lipid, and ash content of whole fish (*P* > 0.05) (Table 4). The crude lipid content was 3.26% and 4.10% lower in the LP-Ly and LL-Ly groups compared with the Control group. The crude protein of whole fish was significantly higher in the LP-Ly group than that in the Control group (*P* < 0.05).

### 3.3. Serum Biochemical Indicators

There were no significant (*P* > 0.05) differences in serum TP, TG, and HDL-C between groups (Table 5). The TC levels in the LP-Ly and LL-Ly groups were significantly lower than those in the Control group (*P* < 0.05). The LDL-C level in the Control group was 2.88% higher than that in the LP-Ly group without significance (*P* > 0.05) difference and was significantly higher than that in the LL-Ly group (*P* < 0.05). The ALT activity was significantly higher in the Control group than in the LP-Ly and LL-Ly groups (*P* < 0.05). The AST activity was significantly lower in the LP-Ly group than in the Control group (*P* < 0.05), but was 6.15% lower in the LL-Ly group than in the Control group (*P* > 0.05).

### 3.4. Digestive Enzyme Activity

The protease and lipase activities in the liver and intestine were significantly higher in the LP-Ly and LL-Ly groups compared to the Control group (*P* < 0.05) (Table 6). The amylase activity in the liver was significantly higher in the LL-Ly group compared to the Control group (*P* < 0.05), while it was 27.41% higher in the LP-Ly group compared to the Control group (*P* > 0.05). Compared to the Control group, the amylase activity in the intestine was not significantly different in the LP-Ly and LL-Ly groups (*P* > 0.05).

### 3.5. Intestinal Microbiota Community Characterization

#### 3.5.1. Sequencing Results and Quality Control

**Figure 1:**
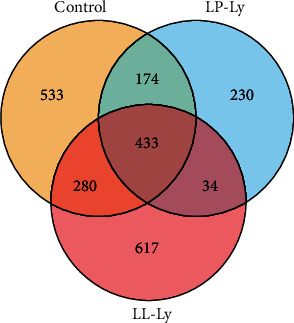
Venn diagrams for comparing OTU distributions in different groups.

A total of 961,428 high-quality sequences with an average length of 441 bp were obtained in this study (Figure 1). The highest and lowest number of unique OTUs was observed in the Control and LP-Ly groups, respectively. The OTU number in the LP-Ly, LL-Ly, and Control groups was 467, 713, and 607, respectively. The OTU number shared by the samples from each treatment group was 433.

#### 3.5.2. *α*-Diversity of Microbial Community Richness

The ACE, Chao1, Shannon, and Simpson were significantly higher in the Control group than in the LP-Ly group (*P* < 0.05) (Table 7). The Shannon and Simpson parameters in the LL-Ly group were not significantly different from those in the Control group (*P* > 0.05), but the ACE and Chao1 parameters in the LL-Ly group were significantly lower than those in the Control group (*P* < 0.05).

**Figure 2:**
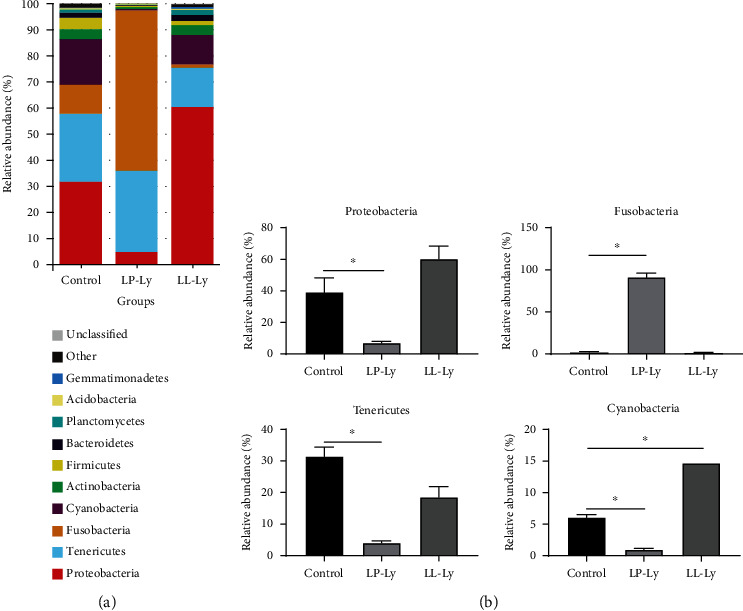
Structure and composition of the intestinal bacterial community of juvenile largemouth bass fed different diets at the phylum level (a). The top 4 microbial communities were analyzed at the phylum level (b). Shoulder scale ∗ indicates the group has significant difference (*P* < 0.05) compared to the Control group.

The top 10 dominant phylum of largemouth bass gut microbes were Proteobacteria, Tenericutes, Fusobacteria, Cyanobacteria, Actinobacteria, Firmicutes, Bacteroidetes, Planctomycetes, Acidobacteria, and Gemmatimonadetes (Figure 2(a)). The top 4 phyla of the fish intestinal flora were further analyzed (Figure 2(b)). Compared to the Control group, the LP-Ly group showed a significant increase in Fusobacteria abundance (*P* < 0.05), but a significant decrease in the abundance of Proteobacteria, Tenericutes, and Cyanobacteria (*P* < 0.05). Compared to the Control group, the LL-Ly group showed a significant increase in Cyanobacteria abundance (*P* < 0.05), while the abundance of Fusobacteria, Proteobacteria, and Tenericutes was not significantly different between groups (*P* > 0.05).

**Figure 3:**
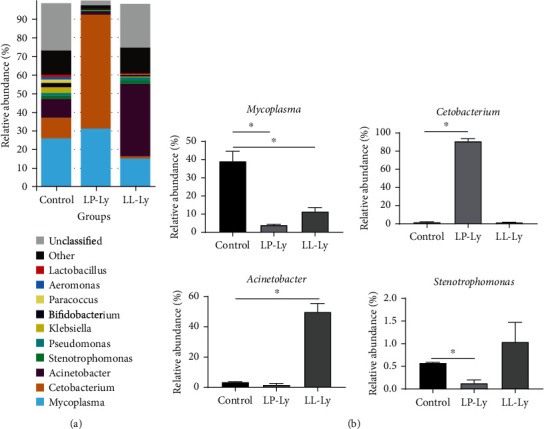
Structure and composition of the intestinal bacterial community of juvenile largemouth bass fed different diets at the genus level (a). The top 4 microbial communities were analyzed at the genus level (b). Shoulder scale ∗ indicates the group has significant difference (*P* < 0.05) compared to the Control group.

The top 10 dominant genera in the intestine were *Mycoplasma*, *Cetobacterium*, *Acinetobacter*, *Stenotrophomonas*, *Klebsiella*, *Bifidobacterium*, *Pseudomonas*, *Paracoccus*, *Lactobacillus*, and *Aeromonas* (Figure 3(a)). Further analysis of the top 4 species in the intestinal flora is showed in Figure 3(b). Compared to the Control group, the LP-Ly group showed significantly reduced abundance of *Mycoplasma* and *Stenotrophomonas* (*P* < 0.05), but significantly higher *Cetobacterium* abundance (*P* < 0.05). Compared to the Control group, the abundance of *Acinetobacter* was significantly higher (*P* < 0.05), but the abundance of *Mycoplasma* was significantly lower (*P* < 0.05) in the LL-Ly group.

#### 3.5.3. Functional Prediction of the Intestinal Flora

**Figure 4:**
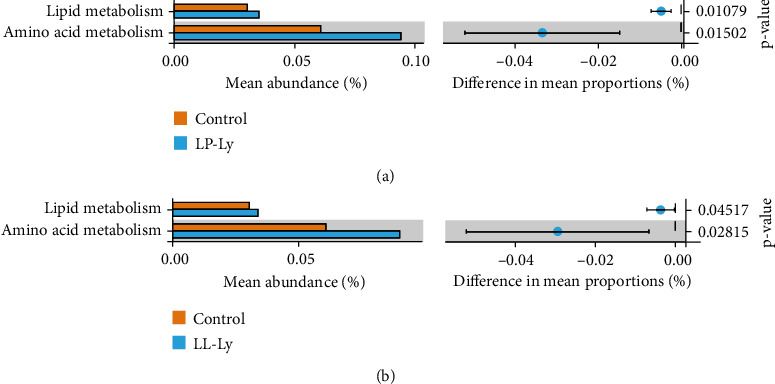
Prediction of the function of metabolic pathways for the intestinal flora of largemouth bass.

The KEGG functional predictions of the intestinal flora of largemouth bass fed the three diets were analyzed using Tax4Fun from SILVA annotations of 16s sequences (Figure 4). Both lipid and amino acid metabolisms in groups LP-Ly and LL-Ly were significantly higher than those in the Control group (*P* < 0.05).

### 3.6. Hepatic Lipid Metabolism-Related Enzymes

The activities of LPL, HSL, CPT-1, FAS, and ACC in the liver of largemouth bass fed the LP-Ly and LL-Ly diets were significantly higher than those in the Control group (*P* < 0.05) (Table 8).

### 3.7. Hepatic Lipid Metabolism-Related Gene Expression

**Figure 5:**
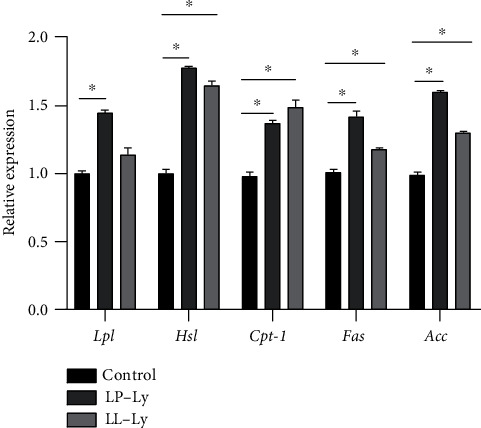
Relative expression of genes related to lipid metabolism in juvenile largemouth bass fed the experimental diets. Shoulder scale ∗ indicates the group has significant difference (*P* < 0.05) compared to the Control group.

The expression of lipoprotein lipase gene (*lpl*), hormone-sensitive lipase gene (*hsl*), fatty acid synthase gene (*fas*), acetyl coenzyme A carboxylase gene (*acc*), and carnitine palmitoyltransferase 1 gene (*cpt-1*) was significantly higher in the LP-Ly group than in the Control group (*P* < 0.05) (Figure 5). Compared to the Control group, the expression of *hsl*, *fas*, *acc*, and *cpt-1* was significantly higher (*P* < 0.05) in the LL-Ly group (*P* > 0.05).

## 4. Discussion

Juvenile animals may secrete insufficient bile salts and lipase, which results in a low capacity of lipid digestion and absorption. Supplementing exogenous emulsifiers in the diet is one of the strategies to improve lipid and energy utilization of the young animals. Previous studies have revealed that the dietary phospholipids significantly increased the growth performance of common carp larvae (*Cyprinus carpio*) [26], blunt snout bream fingerlings [27], large yellow croaker larvae [6], and turbot (*Scophthalmus maximus*) [19, 28]. Dietary lysophospholipid supplementation also promoted the growth performance and carcass yield of the broilers [13]. In this experiment, adding 1 g·kg^−1^ lysophospholipids in low-crude lipid or low-crude protein diets did not significantly affect the growth performance of largemouth bass. There was a decrease trend in body lipid content and an increase trend in crude protein content with lysophospholipid supplementation. This result was similar to that observed in heterozygous silver carp (*Carassius auratus gibelio*) [29], amberjack (*Seriola dumerili*) [30], large yellow croaker [6], and blunt snout bream (*Megalobrama amblycephala*) [27]. It has been reported that dietary lysophospholipid induced an increase in protein synthesis, increased cell size, increased atrial natriuretic factor (ANF) expression, and activated mitogen-activated protein (MAP) kinases [31, 32]. Addition of lysophospholipids to turbot feed aided digestion of dietary lipid and promoted the lipolytic gene expression [33].

Studies with red-spotted grouper (*Epinephelus akaara*) showed that reductions in serum TG and TC levels were found to be associated with reductions in protein and lipid content in the diet [34]. In this experiment, there were no significant differences in serum TG level between the groups, suggesting that a relative decrease in protein or lipid content in feeds supplemented with lysophospholipids did not affect the serum TG level. HDL-C plays an important role in transporting TC and free fatty acids (FFA) from peripheral tissues to hepatocytes for metabolism, while LDL-C transfers TC to peripheral tissue cells [35, 36]. Following lysophospholipid supplementation, the HDL-C level was not affected compared to the control, while the LDL-C level was significantly lower in the LL-Ly group. In this experiment, the lower serum LDL-C and TC levels in group LL-Ly may suggest that lysophospholipid facilitated the transport of LDL-C to liver metabolism. Notably, the structural specificity of lysophospholipids shows a number of nutritional advantages. It affects the composition and function of lipoproteins *in vivo* through different pathways [37]. Studies have shown that n-3 PUFA-rich phospholipids from krill oil can reduce TC, LDL-C, and TG in nonhuman primates [38]. Lysophospholipid helps to improve digestion, transport, and absorption of dietary lipids, thereby improving lipid deposition and energy efficiency and reducing serum levels of TC and LDL-C [39]. In addition, phospholipids can interact with the membranes of intestinal cells and reduce their ability to absorb cholesterol [40]. Lower activities of AST and ALT in serum usually indicate healthy status of the liver [41]. In this study, the lower AST and ALT activities were found in the serum of fish fed the LP-Ly or LL-Ly diets, suggesting that lysophospholipids could maintain the liver health in largemouth bass consuming diets with low protein or lipid levels.

In the present experiment, significantly higher amylase, lipase, and protease activities in the liver and intestine were found in the LP-Ly and LL-Ly groups, which was consistent with the results in carp [42] and Caspian brown trout (*Salmo trutta Caspius*) [43]. Lysophospholipid with the better emulsification can stimulate bile secretion from the gallbladder and directly increase the contact area between celiac particles and digestive enzymes, which relatively increases the substrate concentration and then enhances the digestive enzyme activity.

The complex microbial ecosystem in the gut exhibits a vital role in the nutrition and health of the host [44, 45]. Indeed, the composition of the intestinal flora can be influenced by factors such as feed and environment [46, 47]. Iced trash fish and artificial diets could affect the diversity and composition of gut microbes in largemouth bass [48, 49]. This study showed that reducing feed protein levels with addition of lysophospholipids significantly reduced the diversity of the gut flora of largemouth bass, which however could not be necessarily negative for the fish growth performance.

Proteobacteria, Fusobacteria, and Firmicutes are widespread in the gut of aquatic organisms such as Nile tilapia (*Oreochromis niloticus*) [50], Atlantic salmon (*Salmo salar*) [51], and hybrid grouper [52]. Fusobacteria produces the short-chain fatty acid butyrate [53], which provides energy to gastrointestinal cells, increases mucus secretion, and has some growth-promoting effects [54]. In this experiment, there was no significant change in growth performance in the LP-Ly group compared to the Control group, probably due to the fact that lysophospholipids contribute to the Fusobacteria proliferation and make more butyrate in the intestine of largemouth bass. Cyanobacteria are potentially pathogenic, but Cyanobacteria in low abundance promotes growth and in high abundance produces hepatotoxic microcystins that may be harmful to aquatic animals [55, 56]. The cyanobacteria abundance in this experiment showed a significant decrease in the LP-Ly group but a significant increase in the group LL-Ly. According to the growth performance of these two groups, cyanobacteria could not show negative effects. *Mycoplasma* spp. can cause inflammatory responses [57]. The abundance of *Mycoplasma* was significantly lower in the LP-Ly and LL-Ly groups compared to the Control group. This suggests that the addition of lysophospholipids at the low-protein or low-lipid diets may reduce inflammation in fish to some extent by reducing the abundance of *Mycoplasma* in the intestine. The present study showed that fish fed the LP-Ly diet had increased abundance of *Cetobacterium*, which is protease-producing bacteria that are thought to be involved in peptone fermentation and protein metabolism [58, 59]. Interestingly, this is corroborated by the higher protein content of fish body in group LP-Ly. In addition, *Acinetobacter* has been associated with disease infections in tilapia and humans [60, 61], and the results of the present study showed that its abundance was significantly higher in the LL-Ly group, which may increase the risk of disease infection in fish.

The addition of lysophospholipids can promote protein metabolism and energy metabolism in broilers [62]. In the present experiment, functional predictions of the gut microbiota also indicate that the amino acid metabolism and lipid metabolism were significantly upregulated in the LP-Ly and LL-Ly groups compared to the Control group. Lysophospholipid mechanically promotes the germination of encapsulated vesicles (COPII), which are required for amino acid penetration to be packaged into transport vesicles *in vitro* [63, 64]. Functional diversity of gut microbiota can reflect the influence of gut microbiota on the metabolic processes of host organism [25]. The results of hepatic lipid metabolizing enzyme activity and the relevant gene expression in largemouth bass also indicate that low-nutrient diets supplemented with lysophospholipid contributed to enhancement of hepatic lipid metabolism. The function of intestinal flora is coordinated with the nutrient metabolism in the fish liver.

Previous studies have demonstrated that dietary phospholipids can affect lipid metabolism in fish at the transcriptional level [5]. Therefore, the expression of genes involved in the regulation of lipid metabolism was investigated, in order to explore the mechanisms by which phospholipids induce lipid metabolism in the liver. LPL and HSL are two key enzymes and serve an important role in hepatic lipolysis catabolism of fish [65], of which the activities generally increase with the increase of dietary lipid content [65, 66]. However, the present study showed that the activities and mRNA expression of LPL and HSL increased with the addition of lysophospholipid when the dietary lipid or protein content decreased, which is similar to the findings in broiler chickens [67]. These changes may be related to the increased emulsification of lipids by lysophospholipids [68, 69], which accelerates lipolysis, provides energy, and allows better protein deposition in the body. CPT1 is the rate-limiting enzyme in fatty acid oxidation and the activity of CPT1 is closely related to lipid catabolism [70]. The addition of phospholipids had no significant effect on CPT1 enzyme activity in large yellow croaker [71], but a certain amount of egg yolk lecithin could significantly increase ACC enzyme activity and gene expression in green mud crab [72]. In this study, the enzyme activity and mRNA expression of CPT1 and ACC were significantly higher in low dietary protein and lipid groups supplemented with lysophospholipids. This may be because that lysophospholipids can activate the AMPK*α*-ACC-CPT signaling pathway and increase fatty acid *β*-oxidation in hepatocytes, thus promoting fatty acid catabolism [18]. The *fas* gene had higher expression level in the LP-Ly and LL-Ly groups, showing that lysophospholipid affected the hepatic lipid metabolism in multiple ways. The addition of phospholipids to the diet significantly reduces the lipid accumulation in mammals, which is caused by the downregulation of *fas* expression [73, 74], and similar results have been observed in large yellow croaker [5] and hybrid snakehead [75]. However, the expression of adipogenesis-related genes (such as *fas* and *acc*) of the hybrid grouper was not significantly affected by the dietary phospholipids [7]. In this study, the hepatic lipolysis may be affected at higher levels than lipid synthesis, which facilitates energy supply. These results corresponded to the decrease in body lipid content and increase in body protein content, as well as functional predictions of the gut microbiota.

In conclusion, under the conditions of this feeding trial, supplementation of lysophospholipids in diets with reduced levels of crude protein or crude lipid had no negative impact on the growth performance of largemouth bass compared to diets with normal protein and lipid levels. However, it enhanced the lipid metabolism, alleviated the liver damage, and regulated the structure and diversity of the intestinal flora.

## Figures and Tables

**Table 1:** Formulation and proximate composition of experimental diets.

	Unit price ($/ton)	Control	LP-Ly	LL-Ly
Ingredients (g/kg)				
Brown fish meal	1937.72	500	400	500
Chicken meal	1244.62	100	100	100
Soybean meal	648.39	104	170	104
Fermented soybean meal	715.47	50	50	50
Plasma protein powder	1416.03	20	20	20
Wheat gluten flour	1997.35	15	31	15
Tapioca starch	894.34	40	40	40
Wheat flour	581.32	61	70	70
Seaweed powder	1788.67	20	20	20
Fish oil	2086.78	50	50	40
Soybean oil	1802.08		8	
Vitamin mix^1^	4778.15	10	10	10
Mineral mix^1^	477.81	30	30	30
lysophospholipid^2^	5972.69		1	1
Costs^3^ ($/ton)		1528.16	1434.75	1518.49
Total		1000	1000	1000
Proximate composition (%) (dry matter basis)				
Moisture		7.93	8.20	8.13
Crude protein		54.52	52.46	54.43
Crude lipid		11.45	11.36	10.19
Ash		10.64	10.33	10.47

^1^Vitamin premix and mineral premix (Yuehai Brand®) by Guangdong Yuehai Feed Group Co. Ltd., Zhanjiang, China. ^2^Provided by Kemin AquaScience (Zhuhai, China). ^3^Exchange rates are applied: $1 = *￥*6.7089.

**Table 2:** The primers used in real-time PCR.

Gene name	Sequence of primer (5′-3′)	GenBank no.
*lpl*	F: AACCGCAATCCCTCGCC	XM_038715978.1
	R: AAGGTCTGTGTTTCTGAGTTGA	
*hsl*	F: CACTAACACCCCCACACCAA	XM_038725628.1
	R: CAGAGTCATCCAGCAAGGCA	
*fas*	F: TTACACTGCCACAGCAACCA	XM_038735140.1
	R: TGCCCCTCCTACTACACCTC	
*acc*	F: TAGTCCAGTGCCCATCCTCA	XM_038709737.1
	R: CCAGAAAAGCCCCTCCAGTT	
*cpt-1*	F: AACGGATGGAGGCTTTGACC	XM_038705335.1
	R: CTACACCTGGGACACGACTG	
*β-Actin*	F: AGAGGTTCCGTTGCCCAG	XM_038695351.1
	R: TGCTGTTGTAGGTAGTCTCGT	

*lpl*: lipoprotein lipase; *hsl*: hormone-sensitive lipase; *fas*: fatty acid synthase; *acc*: acetyl-CoA carboxylase; *cpt-1*: carnitine palmitoyltransferase 1; *β-actin*: an internal reference gene.

**Table 3:** Growth performance of largemouth bass fed experimental diets.

Items	Control	LP-Ly	LL-Ly
FBW (g)	39.65 ± 1.91	39.67 ± 0.63	39.67 ± 0.22
WGR (%)	556.50 ± 31.50	555.11 ± 10.00	556.11 ± 3.33
SGR (%)	2.93 ± 0.09	2.92 ± 0.01	2.94 ± 0.01
SR (%/d)	94.17 ± 0.84	95.93 ± 4.07	96.67 ± 3.34
FCR	0.85 ± 0.03	0.83 ± 0.07	0.82 ± 0.06
FR (%)	1.93 ± 0.02	1.92 ± 0.23	1.89 ± 0.12
HSI (%)	2.94 ± 0.14	2.66 ± 0.07	2.76 ± 0.14
VSI (%)	7.34 ± 0.22	6.82 ± 0.13	6.65 ± 0.49
CF	1.83 ± 0.05	2.02 ± 0.04^∗^	1.89 ± 0.05
PDR (%)	38.90 ± 3.71	41.24 ± 1.42	42.11 ± 3.65

Note: FBW: final weight body; WGR: weight gain; SGR: specific growth rate; SR: survival rate; FCR: feed conversion ratio; FR: feeding ratio; HSI: hepatosomatic index; VSI: viscerosomatic index; CF: condition factor; PDR: protein deposition rate. Shoulder scale ∗ indicates the group has significant difference (*P* < 0.05) compared to the Control group.

**Table 4:** Proximate composition of largemouth bass fed with diets (dry weight).

Index (%)	Control	LP-Ly	LL-Ly
Moisture	72.14 ± 0.40	72.17 ± 0.93	72.35 ± 0.18
Crude protein	59.57 ± 0.43	61.45 ± 0.05^∗^	60.67 ± 0.34
Crude lipid	20.22 ± 0.62	19.56 ± 0.50	19.39 ± 0.54
Ash	14.87 ± 0.15	14.50 ± 0.00	15.33 ± 0.17

Shoulder scale ∗ indicates the group has significant difference (*P* < 0.05) compared to the Control group.

**Table 5:** Serum biochemical index of juvenile largemouth bass fed the experimental diets.

Index	Control	LP-Ly	LL-Ly
TP (mmol/L)	8.11 ± 0.19	7.35 ± 0.54	7.43 ± 0.49
TG (mmol/L)	2.41 ± 0.26	2.07 ± 0.21	2.12 ± 0.07
TC (mmol/L)	6.80 ± 0.08	6.09 ± 0.07^∗^	5.17 ± 0.21^∗^
HDL (mmol/L)	7.21 ± 0.42	6.36 ± 0.73	5.94 ± 0.42
LDL (mmol/L)	3.47 ± 0.24	3.37 ± 0.10	2.07 ± 0.02^∗^
AST (mU/mL)	9.76 ± 0.16	8.53 ± 0.29^∗^	9.16 ± 0.05
ALT (mU/mL)	11.68 ± 0.23	8.64 ± 0.59^∗^	8.28 ± 0.43^∗^

Note: TP: total protein; TG: triglycerides; TC: total cholesterol; HDL: high-density leptin cholesterol; LDL: low-density lipoprotein cholesterol; AST: aspartate aminotransferase; ALT: alanine aminotransferase. Shoulder scale ∗ indicates the group has significant difference (*P* < 0.05) compared to the Control group.

**Table 6:** Digestive enzyme activity of juvenile largemouth bass fed the experimental diets.

Index (mg/g·prot)	Control	LP-Ly	LL-Ly
Liver			
Lipase	1.04 ± 0.03	1.17 ± 0.03^∗^	1.27 ± 0.03^∗^
Protease	4.39 ± 0.03	6.82 ± 0.08^∗^	4.69 ± 0.08^∗^
Amylase	1.97 ± 0.02	2.51 ± 0.21	3.38 ± 0.08^∗^
Intestinal			
Lipase	1.93 ± 0.05	2.34 ± 0.10^∗^	2.86 ± 0.09^∗^
Protease	0.87 ± 0.01	1.03 ± 0.01^∗^	1.01 ± 0.01^∗^
Amylase	4.78 ± 0.13	5.20 ± 0.18	5.97 ± 0.45

Shoulder scale ∗ indicates the group has significant difference (*P* < 0.05) compared to the Control group.

**Table 7:** The *α*-diversity of microbial communities of juvenile largemouth bass fed the experimental diets.

Diets	Shannon	Simpson	Chao1	Ace
Control	6.68 ± 0.28	0.97 ± 0.01	2321.90 ± 68.46	2435.27 ± 125.64
LP-Ly	4.40 ± 0.19^∗^	0.91 ± 0.01^∗^	1210.28 ± 241.13^∗^	1232.90 ± 237.54^∗^
LL-Ly	6.35 ± 0.35	0.96 ± 0.01	1849.42 ± 91.85^∗^	1888.51 ± 93.38^∗^

Shoulder scale ∗ indicates the group has significant difference (*P* < 0.05) compared to the Control group.

**Table 8:** Liver lipid metabolism-related enzymes of the juvenile largemouth bass fed the experimental diets.

Index (ng/g·prot)	Control	LP-Ly	LL-Ly
Lipolysis			
LPL	127.09 ± 1.84	165.62 ± 1.73^∗^	159.62 ± 1.54^∗^
HSL	67.26 ± 1.52	147.61 ± 3.44^∗^	145.32 ± 3.92^∗^
CPT-1	138.22 ± 3.84	171.24 ± 4.73^∗^	188.42 ± 1.76^∗^
Lipid synthesis			
FAS	8.44 ± 0.06	10.73 ± 0.11^∗^	10.96 ± 0.05^∗^
ACC	38.38 ± 0.67	48.78 ± 1.60^∗^	51.94 ± 1.65^∗^

Note: LPL: lipoprotein lipase; HSL: hormone-sensitive lipase; CPT-1: carnitine palmitoyltransferase 1; FAS: fatty acid synthase; ACC: acetyl-CoA carboxylase. Shoulder scale ∗ indicates the group has significant difference (*P* < 0.05) compared to the Control group.

## Data Availability

The processed data required to reproduce these findings cannot be shared at this time as the data also forms part of an ongoing study.
